# Vitamin D Status of Infants in Northeastern Rural Bangladesh: Preliminary Observations and a Review of Potential Determinants

**DOI:** 10.3329/jhpn.v28i5.6154

**Published:** 2010-10

**Authors:** Daniel E. Roth, M. Rashed Shah, Robert E. Black, Abdullah H. Baqui

**Affiliations:** ^1^ Department of International Health, Johns Hopkins Bloomberg School of Public Health, 615 North Wolfe Street, Baltimore, MD 21205, USA; ^2^ Projahnmo, Sylhet, Bangladesh

**Keywords:** Risk factors, 25-hydroxyvitamin D, Vitamin D, Vitamin D deficiency, Bangladesh

## Abstract

Vitamin D deficiency is a global public-health concern, even in tropical regions where the risk of deficiency was previously assumed to be low due to cutaneous vitamin D synthesis stimulated by exposure to sun. Poor vitamin D status, indicated by low serum concentrations of 25-hydroxyvitamin D [25(OH)D], has been observed in South Asian populations. However, limited information is available on the vitamin D status of young infants in this region. Therefore, to gain preliminary insights into the vitamin D status of infants in rural Bangladesh, 25(OH)D was assessed in a group of community-sampled control participants in a pneumonia case-control study in rural Sylhet, Bangladesh (25°N) during the winter dry season (January-February). Among 29 infants aged 1-6 months, the mean 25(OH)D was 36.7 nmol/L [95% confidence interval (CI) 30.2-43.2]. The proportion of infants with vitamin D deficiency defined by 25(OH)D <25 nmol/L was 28% (95% CI 10-45), 59% (95% CI 40-78) had 25(OH)D<40 nmol/L, and all were below 80 nmol/L. From one to six months, there was a positive correlation between age and 25(OH)D (Spearman=0.65; p=0.0001). Within a larger group of 74 infants and toddlers aged 1-17 months (cases and controls recruited for the pneumonia study), young age was the only significant risk factor for vitamin D deficiency [25(OH)D <25 nmol/L]. Since conservative maternal clothing practices (i.e. veiling) and low frequency of intake of foods from animal source (other than fish) were common among the mothers of the participants, determinants of low maternal-infant 25(OH)D in Bangladesh deserve more detailed consideration in future studies. In conclusion, the vitamin D status in young infants in rural Sylhet, Bangladesh, was poorer than might be expected based on geographic considerations. The causes and consequences of low 25(OH)D in infancy and early childhood in this setting remain to be established.

## INTRODUCTION

Throughout most of the previous century, vitamin D deficiency and rickets were predominantly perceived as problems of industrialized countries at northern latitudes, where insufficient exposure to sun and intake of vitamin D were linked to inadequate intestinal absorption of calcium and impaired skeletal mineralization (1). However, since vitamin D status has become readily estimable based on serum/plasma 25-hydroxyvitamin D concentration [25(OH)D] (2), epidemiologic research aimed at identifying previously-uncharacterized at-risk populations and characterizing novel disease associations with vitamin D status has been greatly facilitated. Thus, vitamin D deficiency has re-emerged as a global public-health concern and is now presumptively linked to a range of infectious, inflammatory and neoplastic diseases throughout the life course and around the world (1).

Low 25(OH)D is surprisingly common in South Asia, where systemic vitamin D deficits would be expected to be prevented by cutaneous vitamin D synthesis stimulated by exposure to sun at relatively low latitudes (3). Few studies on vitamin D status in infancy have been conducted in South Asia (Table 1). In Bangladesh, two published reports on childhood 25(OH)D are available but neither reported 25(OH)D in early infancy (Table 1).

**Table 1.** Summary of studies of vitamin D status of infants and children aged 1 month to <5 years in Bangladesh, Pakistan, and India

Study	Participants	Location	Latitude	Season	No.	Mean 25(OH)D (nmol/L)	Categorical descriptions of vitamin D status
Combs *et al*., 2008 (9)	Aged 1-5 years; no clinical signs of rickets	Chakaria subdistrict, Cox's Bazar, Bangladesh	21°N	Unknown	158	68.3	25(OH)D <25 nmol/L in 6% (10/158) 25(OH)D <37.5 nmol/L in 21% (33/158)
Fischer *et al*., 1999 (10)	Cases in case-control study of rickets; aged 10-120 months	Chakaria subdistrict, Bangladesh	21°N	Unknown	14	50	Range 17.5-162.5 nmol/L 25(OH)D <35 nmol/L in 2/14 (14%).
	Controls	Chakaria subdistrict, Bangladesh	21°N	Unknown	13	62.5	Range 55-87.5 nmol/L None with 25(OH)D <35 nmol/L
Atiq *et al*., 1998 (11)	Aged 6 weeks-11 months; immunization clinic	Karachi, Pakistan	25°N	November-March	23	24.5	25(OH)D <25 nmol/L in 52% (throughout the year)
				April-October	48	40.7	
Agarwal *et al*., 2002 (12)	Aged 9-24 months; high-pollution area	Morigate, Delhi, India	28°N	March-April	26	31	25(OH)D <30 nmol/L in 35%
	Aged 9-24 months; low-pollution area	Gurgaon, Delhi, India	28°N	March-April	31	68	25(OH)D <30 nmol/L in 0%
Bhalala *et al*., 2007 (13)	Healthy breastfed infants aged 3 months	Mumbai, India	18°N	Throughout the year	35	45.5	25(OH)D <62.5 nmol/L in 80%
Tiwari and Puliyel, 2004 (14)	Aged 9-30 months living in impoverished neighbourhoods	Sundernagari, Delhi, India	28°N	January	47	96.3	25(OH)D <35 nmol/L in 2%
		Rajiv Colony, Delhi, India	28°N	February	49	23.8	25(OH)D <35nmol/L in 82.9%
		Rajiv Colony, Delhi, India	28°N	August	48	17.8	25(OH)D <35 nmol/L in 84.0%
		Gurgaon, Delhi, India	28°N	August	52	19.2	25(OH)D <35 nmol/L in 82.0%
Wayse *et al*., 2004 (15)	Healthy controls in case-control study; aged <5 years	Indapur, India	17°N	May-June	70	38.4	25(OH)D <22.5 nmol/L in 31% 25(OH)D <50 nmol/L in 61%

Knowledge of the vitamin D status of young children and infants is needed to design studies targeting the aetiologic mechanisms and potential health implications of deficiency. A case-control study on the association between acute lower respiratory tract infection (ALRI) and vitamin D status in infants and young children conducted in Zakiganj subdistrict of Sylhet district in Bangladesh, during January-February 2008, provided an opportunity to gain preliminary insights into the vitamin D status of infants in northeastern rural Bangladesh (4). Here, we aimed to describe the vitamin D status of the source population and briefly review the potential determinants of low infant 25(OH)D in this setting.

## MATERIALS AND METHODS

### Setting

Zakiganj subdistrict (upazila) is in Sylhet district of northeastern Bangladesh (25°N), on the border with India. This region has low average household income and maternal literacy and has limited access to healthcare compared to neighbouring subdistricts in Sylhet (5). The study was facilitated by strong existing partnerships with local community organizations, the subdistrict health complex, and the Ministry of Health and Family Welfare, based on an ongoing collaborative neonatal health intervention trial infrastructure (Projahnmo) (6).

### Participants

ALRI cases who met a clinical definition of ALRI were recruited from among infants and young children, aged one month to two years, admitted to the Zakijang subdistrict hospital. Control participants were selected by sampling from among children who lived in the same villages as the cases, were matched to a case on age (±2 months) and gender, and had no signs of ALRI at recruitment or reported past history of ALRI/pneumonia. To identify controls, a rapid household census was conducted in the village of residence of each case participant to generate a list of eligible controls aged 1-23 months. In an order based on closeness in age to the index case, caregivers of the listed children were approached until a control participant was recruited, consent was obtained, and a blood specimen was collected. If an eligible control was not enrolled, the census and eligible control identification process was repeated in the nearest neighbouring village. Some recruited children not considered eligible for the primary case-control study were included in the present analysis. The major reasons for this difference were that children enrolled during a one-week pilot phase were not included in the case-control study but were included here, and the strict requirement that gross haemolysis be absent on visual inspection of serum specimens included in the case-control study was relaxed for the present analysis. This latter decision was made based on post-hoc findings that the mean 25(OH)D of grossly haemolyzed specimens was only slightly and non-significantly lower than that of non-haemolyzed serum specimens (difference of means=3.6 nmol/L, p=0.335, after adjustment for case-control status).

### Collection of data

Caregivers (mothers) of participants were administered a questionnaire that addressed selected infant, maternal and household characteristics potentially associated with vitamin D status. Maternal intake of foods from animal sources, which included potential sources of vitamin D and rich sources of calcium, was assessed based on the reported frequency of consumption of food items/categories over the seven days preceding enrollment. Participants were further categorized as to whether the mother had consumed each food item/category at least once in the preceding seven days. Weight of infant was the average of two measurements recorded to the nearest 0.1 kg (Seca 354 infant scale), and length was the average of two measurements, to the nearest 0.5 cm (Seca 210 measuring mat). Gender-specific weight-for-age (WA), length-for-age (LA), and body mass index (BMI) z-scores were calculated according to the growth standards of the World Health Organization (7). According to convention, participants with z-score values of less than -2 for each of the anthropometric indices were considered to have stunting (LA), underweight (WA), and low body mass index (BMI). Since reliable information on gestational age at birth was unavailable, anthropometric measures were interpreted under the assumption of term gestation.

A venous blood specimen was collected by standard methods, separated into serum aliquots, and stored at -20 °C or less. At the completion of the study, sera were shipped to the laboratory of Dr. Bruce Hollis, Medical University of South Carolina, Charleston, USA, for measurement of the total serum 25(OH)D concentration by radio-immunoassay (8).

### Outcomes

The primary outcome of the study was the estimated mean 25(OH)D among infants and children aged one month to two years, in the referral area of Zakiganj subdistrict hospital, based only on the healthy control participants sampled from the community. Since most controls (29/35) from whom blood samples were obtained were aged less than six months, the analysis was focused on this age subgroup. Estimates of the prevalence of vitamin D deficiency were based on the proportion of participants with 25(OH)D lower than pre-specified cut-offs (<25 nmol/L, <40 nmol/L, and <80 nmol/L).

As a secondary exploratory analysis, observations relating to infant, maternal or household factors that potentially influenced vitamin D status were drawn from the complete sample of 74 participants (cases and controls) in whom 25(OH)D was measured. Comparisons were made between groups of participants categorized by vitamin D status using a 25-nmol/L cut-off. ALRI cases were included in this analysis to maximize the size of the available sample of infants in whom risk factors could be explored. However, in the primary case-control study, the mean 25(OH)D was significantly lower among ALRI cases compared to controls (4).

### Statistical analysis

The distribution of 25(OH)D among control participants aged less than six months (n=29) was described by its mean and 95% confidence interval (CI), standard deviation (SD), median and interquartile range (IQR), and the proportions (and 95% CIs) of participants with 25(OH)D less than each of the cut-off values. Other than age and 25(OH)D, which were compared across groups by one-way analysis of variance, the statistical significance of bivariate associations between the vitamin D status and the maternal, infant or household characteristics in the entire sample (n=74) was assessed by non-parametric tests, including chi-square tests, Mann-U Whitney tests, and Spearman's rank correlation coefficient. Given that the determinants of vitamin D status may differ across age-groups, associational analyses were repeated among participants aged less than six months. Analyses were performed using the Stata software (version 10.1) (Stata Corporation, College Station, TX, USA). By convention, the p values of less than 0.05 were considered significant.

### Ethics

Caregivers provided signed permission before enrollment. The Institutional Review Board of the Johns Hopkins Bloomberg School of Public Health and the ethics committee of the Bangladesh Institute for Child Health at the Dhaka Shishu Hospital, Bangladesh, approved the study.

## RESULTS

### Characteristics of study participants

Serum 25(OH)D was measured in the 74 participants (39 ALRI cases and 35 controls) during the study. Of 58 potential controls identified in village censuses and approached for participation, 14 were not enrolled due to parental refusal, six due to inability to bring the child to the hospital for study procedures, and three due to history of ALRI. The subgroup of 29 community control participants, aged 1-6 months, consisted mainly of boys (a result of the male predominance among the ALRI cases to whom the controls were sex-matched), with maternal and household characteristics typical of rural Sylhet (Table 2).

**Table 2.** Characteristics of a sample of infants aged 1-6 months in rural Sylhet district, Bangladesh

Characteristics	Mean±SD or no. (%)
No.	29
Age (days)	71±32
Boys	25 (86)
Age (years) of mothers	23.8±4.2
Mother attended any school	19 (66)
Father attended any school	18 (62)
Family owns their own home	19 (66)
Lives in a house with components made of manufactured materials (i.e. cement, brick, tin)	
Floor	1 (3)
Walls	9 (31)
Roof	27 (93)
Occupation of primary income-earner of household	
Day labourer	15 (52)
Farmer on leased land	2 (7)
Land owner	2 (7)
Non-agricultural business-owner	5 (17)
Salaried non-agricultural job	5 (17)
Muslim religion	27 (93)
Mother wears a burka when in public	27 (93)
Exclusively breastfeeding	23 (79)
Anthropometric measures	
Weight-for-age z-score	-1.58±1.29
Length-for-age z-score	-1.08±1.57
Body mass index z-score	-1.40±1.67

SD=Standard deviation

### Vitamin D status of infants aged 1-6 months

**Fig. 1.**
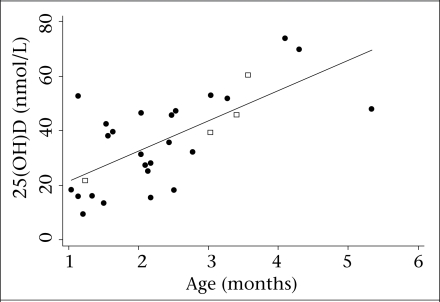
Association between serum 25-hydroxyvitamin D concentration and age in 29 community-sampled infants aged 1-6 months in Sylhet district, Bangladesh

Serum 25(OH)D ranged from 9.5 to 73.9 nmol/L among the community-sampled infants aged 1-6 months (Fig. 1). Their mean 25(OH)D was 36.7 nmol/L (95% CI 30.2-43.2; SD=17.1 nmol/L), and the median was 38.2 nmol/L (IQR 25.5). The proportion of infants with 25(OH)D below each cut-off level was 28% (95% CI 10-45) <25 nmol/L, and 59% (95% CI 40-78) <40 nmol/L; all were below 80 nmol/L. From one to six months, there was a positive correlation between age and 25(OH)D (Spearman ρ=0.65; p=0.0001) (Fig. 1). Among the youngest 22 infants aged one to less than three months, 36% (95% CI 15-58) had 25(OH)D <25 nmol/L, and 73% (95% CI 53-93) had 25(OH)D <40 nmol/L.

Boys are represented by filled circles and girls by hollow squares; the solid line is a linear leastsquares fit line for boys and girls combined

### Characteristics associated with low 25(OH)D

**Fig. 2.**
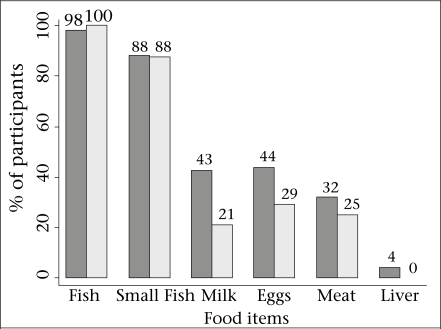
Percentage of the study participants whose mothers consumed the specified food item/category at least once during the preceding 7 days, among those participants with 25(OH)D ≥25 nmol/L (dark bars; n=50), and those with 25(OH)D <25 nmol/L (light gray bars; n=24)

Of the 74 participants (cases and controls) aged 1-17 months, the mean 25(OH)D was 32.6 nmol/L (95% CI 29.1-36.2), the highest 25(OH)D in any participant was 73.9 nmol/L, 24 (32%) had 25(OH)D <25 nmol/L, and 52 (70%) had 25(OH)D <40 nmol/L. In this group, age was the only factor that was significantly associated with vitamin D deficiency defined by 25(OH)D <25 nmol/L (Table 3). However, deficient infants/toddlers tended to be of lower socioeconomic status based on household ownership and housing materials and were somewhat more likely to be stunted and at low BMI (Table 3). Although there were no significant associations between maternal dietary intake and infant/toddlers’ 25(OH)D (data not shown) or vitamin D deficiency (Table 3), a higher proportion of the mothers of infants with 25(OH)D ≥25 nmol/L consumed milk, meat, and eggs on at least one occasion during the week preceding the interview (Fig. 2). Results of analyses of associations of vitamin D status with potential risk factors were similar when repeated in a group restricted to participants aged less than six months (data not shown).

Fish referred to any fish whereas small fish referred to those typically consumed with bones (e.g. *mola*); meat referred to flesh (i.e. poultry, beef, or mutton)

**Table 3.** Comparison of selected characteristics of infants and toddlers categorized by vitamin D status in Sylhet, Bangladesh

Characteristics	25(OH)D <25 nmol/L	25(OH)D ≥25 nmol/L	p value
No.	24	50	
Mean 25(OH)D±SD	16.3±5.8	40.5±11.8	<0.001
Age (mean days±SD)	71.3±43.4	136.6±126.7	<0.001
<6 months	23 (96)	39 (78)	0.051
Boys	21 (88)	40 (80)	0.427
Birth-order (median±IQR)	2±4.5	2±5	0.455
Anthropometric indicators			
Underweight	11 (46)	25 (48)	0.861
Stunted	10 (42)	16 (32)	0.415
Low body mass index	14 (58)	20 (40)	0.138
Age of mothers (mean years±SD)	23.7±5.6	24.8±5.3	0.410
Years of schooling of mother (median±IQR)	1.5±6.5	5±6	0.613
Years of schooling of father (median±IQR)	0±5	4±5	0.177
Family owns their own homestead	14 (58)	32 (64)	0.638
Manufactured housing materials (i.e. cement, brick, or tin)			
Floor	0	3 (6)	0.221
Walls	6 (25)	16 (32)	0.537
Roof	20 (83)	45 (90)	0.411
Child is/was usually swaddled during the first 6 months of life	24 (100)	46 (92)	0.154
Child was deliberately exposed to sunlight before learning to crawl/walk	18 (75)	36 (72)	0.786
Mother's clothing practices when outdoors			
Head usually covered	23 (96)	50 (100)	0.146
Face usually covered	0	0	-
Arms usually covered	24 (100)	48 (96)	0.321
Usually wears burka in public	21 (91)	47 (94)	0.672
Number of meals at which mother reportedly consumed food items from animal source during the past week (median±IQR)			
Any type of fish	13.0±5	14.5±12	0.158
Small fish with bones	6.0±7	6±8	0.561
Milk	0±0	0±7	0.119
Meat	0±0.5	0±2	0.415
Eggs	0±1.5	0±2	0.247
Liver	0±0	0±0	0.324

Figures in parentheses indicate percentages;

IQR=Interquartile range;

SD=Standard deviation

## DISCUSSION

This preliminary study in Sylhet during the winter dry season revealed that the vitamin D status of young infants in rural Bangladesh might be poor enough to put many at risk of rickets and other potential vitamin D-related health consequences. Applying a very conservative definition of vitamin D deficiency [25(OH)D <25 nmol/L], we estimated that about one-third of infants aged 1-6 months may be vitamin D-deficient. To our knowledge, this is the first report of vitamin D status in young infants in Bangladesh. However, the causes and consequences of low 25(OH)D in this setting remain to be determined.

To put these findings in a global context, it is first useful to draw a comparison with what is perhaps the only young infant ‘reference’ group studied at the equator where vitamin D status would be expected to be optimal throughout the year in the absence of dietary supplementation or fortification. In a sample of infants in Oyem, Gabon (1°N), the mean 25(OH)D was 110 nmol/L (SD 43) at birth, 149 nmol/L (SD 54) at three months of age, and 151 nmol/L (SD 64) at six months of age (16). These values suggest a very wide variation but that most infants in that setting were well above the 25(OH)D threshold currently considered optimal (>80 nmol/L) (17). The present findings from Bangladesh were somewhat intermediary between the results of two other studies in South Asia. In Karachi, Pakistan (25°N), 38 breastfed infants aged less than six months had a mean 25(OH)D of 25 nmol/L (18 SD), and 71% of infants (12/17) aged less than three months, had 25(OH)D <40 nmol/L (11). Further south, in Mumbai, India (18°N), 35 breastfed infants at three months of age had a mean 25(OH)D of 49 nmol/L (SD 24), and 51% had values of <37.5 nmol/L (13). In the United Arab Emirates (UAE), at latitude 24°N (about the same as Bangladesh), 78 breastfed term infants aged 1-4 months, born to women with low milk intake and a habitual practice of covering the skin entirely when outdoors, had a median 25(OH)D of only 11.5 nmol/L, and 82% had 25(OH)D <25 nmol/L (18).

In Iowa, USA (41°N), in a longitudinal study of predominantly white infants who were all exclusively breastfed and not receiving supplements, the mean 25(OH)D at about three and half months of age was 33 nmol/L for those assessed in the summer (50% at <27.5 nmol/L) and 17 nmol/L in the winter (79% at <27.5 nmol/L); at approximately six months of age, the mean 25(OH)D increased to 45 nmol/L in the summer (32% at <27.5 nmol/L) but remained at 17 nmol/L for those measured in the winter (82% <27.5 nmol/L) (19). An exogenous vitamin D source is recommended for all infants in North America. So, it is worthwhile noting that, among formula-fed or vitamin D-supplemented infants (mean vitamin D intake of ∼370 IU per day) aged 1-6 months (n=37) enrolled in a hospital-based study during the winter in Alberta, Canada (53°N) (20), the mean 25(OH)D was 78 nmol/L (Roth D *et al*. unpublished observations). Although there was a wide variation (17-152 nmol/L), only one infant aged 1.3 months had 25(OH)D <40 nmol/L, demonstrating the real-world effect of supplementation/fortification policies.

Therefore, these data from rural Bangladesh, in combination with earlier findings from infants in urban Pakistan, India, and UAE, have demonstrated that the vitamin D status of young infants in South Asia and the Middle East may be no better than that of unsupplemented infants at much higher northern latitudes in North America, where guidelines support the provision of routine vitamin D supplementation to all breastfed infants (21,22). This implies that a tropical climate does not necessarily protect against low 25(OH)D in early infancy. Although cutaneous pre-vitamin D3 synthesis is expected to occur year-round in South Asia on the basis of latitude (23), there is a substantial seasonal variation in ultraviolet B irradiance (24). In fact, a seasonal differential in vitamin D status was observed among Pakistani infants (11). Therefore, the representativeness of our data is limited because they reflect vitamin D status during the winter, when ultraviolet radiation exposure is at its nadir, and when cutaneous vitamin D synthesis would be expected to be relatively minimal (24). Moreover, in the Bengal region, the attenuation of actual summer-time ultraviolet radiation exposure due to monsoon cloud-cover (24) may prevent sufficient endowment of vitamin D stores during the summer and, thus, further increase the risk of deficiency during the winter. Our cross-sectional observations suggest that 25(OH)D in Bangladeshi infants may rise within the first few months of life. However, these age-dependent differences may have been confounded by seasonal timing of gestation—a younger age implied that the third trimester coincided with the expected seasonal nadir of vitamin D synthesis, when maternal vitamin D stores might be relatively depleted and, thus, when transfer of vitamin D metabolites to the foetus may have been minimized.

To further explain the apparent ‘vitamin D paradox’ in South Asia (3), a range of hypothetical mechanisms can be proposed (Table 3). Young infants depend almost entirely on the transplacental transfer of vitamin D and 25(OH)D, which explains the consistent association between maternal and cord-blood 25(OH)D (25) and the observation that maternal antenatal vitamin D supplementation augments both maternal and cord-blood 25(OH)D (26). Therefore, the major reason that Bangladeshi infants start life with poor vitamin D stores is low maternal antenatal 25(OH)D, which has been documented in urban and rural Bangladeshi women of reproductive age (27). Islam *et al.* recently studied female workers in a garment factory in Dhaka and speculated that their long day-time hours in indoors, brief exposure to low-intensity sunlight in the early morning, outdoor air pollution, and widespread sunscreen use, in combination with darkly-pigmented skin, may contribute to their poor vitamin D status (mean 25(OH)D of 37 nmol/L, and 15% of the participants had 25(OH)D <25 nmol/L) (28). Conservative dress, including almost complete skin coverage by traditional veils or cloaks, has been emphasized as a contributor to vitamin D deficits in Muslim women in South Asia and the Middle East because it limits cutaneous vitamin D synthesis regardless of the intensity of ambient ultraviolet B (29).

Inferences regarding the determinants of infant/toddlers’ vitamin D status in this study were limited, largely because of the small sample-size and substantial uniformity with respect to selected maternal clothing and dietary practices. We also acknowledge that pooling of ALRI cases and controls to maximize our available sample-size may have led to selection biases. However, the observation that mothers of infants with relatively low 25(OH)D seemed less likely to consume foods from animal sources (other than fish) deserves consideration in future studies. The amount of vitamin D in the local diet is unknown but low calcium intake (or reduced absorption of calcium due to high phytate intake), typical of low-income diets in Bangladesh (30,31), may accelerate 25(OH)D use, leading to relatively-increased vitamin D demands (32). Since the concentration of vitamin D metabolites in breastmilk is determined by maternal vitamin D status, maternal vitamin D deficiency during lactation may cause ongoing deficits in postnatal infants’ vitamin D intake (42); yet, maternal factors cannot entirely account for the persistence of low 25(OH)D among toddlers (Table 1) who experience direct exposure to sun and should be unaffected by the conservative clothing practices of their mothers. Therefore, much remains to be learnt about the determinants of vitamin D status throughout infancy and childhood, particularly where 25(OH)D appears discrepant from that which would be expected based on latitude.

**Table 4.** Hypothetical mechanisms that may contribute to risk of maternal-infant vitamin D deficiency in South Asia

General mechanism	Examples of specific hypothesized mechanisms
Endogenous cutaneous vitamin D production	Conservative clothing practices (33) Limited time spent outdoors Dark skin pigmentation (34) Cloud-cover and outdoor air pollution (12) Geographic/genetic/dietary factors that alter epidermal 7-dehydrocholesterol composition or concentration
Dietary vitamin D intake	Inadequate intake of liver, eggs, fish, or fish oils, or fortified dairy products Diminished natural vitamin D content of farmed fish (35) Reduction in vitamin D content of fish due to cooking (e.g. frying) or processing
Intestinal vitamin D absorption and storage	Nutrient-nutrient interactions or competition, e.g. taurine (36) Inadequate consumption of dietary fatty acid Chronic intestinal inflammation (37) Cholestatic liver disease (and reduced bile acid excretion) Low fat mass, leading to reduced capacity to store vitamin D
Hepatic conversion of vitamin D to 25-hydroyxyvitamin D	Chronic hepatitis or hepatic dysfunction, e.g. aflatoxin exposure (38) Dietary/environmental exposure to P450 cytochrome inhibitors
Destruction or use of 25-hydroxyvitamin D or 1,25-dihydroxyvitamin D	Dietary calcium deficit and/or phytate-rich diet, driving secondary hyperparathyroidism Areca nut consumption (39) Protein-energy malnutrition (40) Nutrient-nutrient interactions, e.g. vitamin A (41) Genetic polymorphisms in genes encoding proteins involved in vitamin D metabolism, e.g. vitamin D receptor, 24-hydroxylase

Despite the current enthusiasm for vitamin D supplementation in the USA (22), the effects of vitamin D deficits during early infancy are still not fully understood, and meaningful inflection points in 25(OH)D-outcome relationships have not been established. The most widely-accepted manifestation of severe vitamin D deficiency in infancy is rickets, the classical childhood metabolic bone disease associated with skeletal hypomineralization and deformities, muscle weakness, and growth impairment (1). Young infants with severe congenital vitamin D deficiency may present with hypocalcaemic tetany or seizures, with absent or subtle skeletal pathology (43–45). Greer noted that there is no clear or consistent association between 25(OH)D and the risk of rickets or other functional outcomes (46). Although 25(OH)D <25 nmol/L is typical of clinically-apparent rickets, early stages of the disease may occur at higher 25(OH)D (∼40 nmol/L) (47), with declines in 25(OH)D occurring as the disease progresses and vitamin D stores are depleted. The frequent occurrence of 25(OH)D >25 nmol/L among toddlers and older children with rickets has been presumptively attributed to dietary calcium deficits (48); however, rickets among breastfed infants with 25(OH)D >25 nmol/L (49) suggests that factors other than calcium intake may be implicated.

In Bangladesh, rickets may be more common than previously thought, based on surveys of lower limb deformities in ambulating children (50,51). Recent data from a nationwide survey suggest that about 0.6% of Bangladeshi children, aged 1-15 years, may have radiologic evidence of rickets, with the highest prevalence in Chittagong and Sylhet divisions (51). The incidence of symptomatic hypocalcaemia secondary to vitamin D deficiency in early infancy is unknown. Some investigators have played down the role of vitamin D in rickets in Bangladesh (10, 52), instead blaming dietary calcium deficits or other mineral deficiencies or excesses, e.g. aluminum (53). However, in case-control studies, dietary calcium intake between rickets-affected and unaffected households did not differ (54) whereas the mean 25(OH)D in cases was significantly lower than in controls (10). A plausible hypothesis is that vitamin D deficiency acts synergistically with other causes of inadequate bone mineralization and that an individual's 25(OH)D concentration below which clinical signs emerge depends on the severity and multiplicity of other genetic and environmental factors.

Beyond rickets, speculation abounds regarding the potential extra-skeletal consequences of suboptimal vitamin D status during foetal development and infancy. The vitamin D receptor has been found within virtually every organ-system (55), and the active metabolite of vitamin D is well-described as a potent mediator of cell proliferation and differentiation, particularly noted for its range of effects on immune function in laboratory models (56). The case-control study for which the data for the present analysis were primarily collected revealed an inverse association between 25(OH)D and the odds of hospitalization for ALRI (4), corroborating the findings in neonates in Turkey (57) and children in India (15). If vitamin D deficiency is confirmed as a risk factor for pneumonia, interventions to improve maternal-infant vitamin D status could reduce the global burden of ALRI, the single most important cause of early childhood death in the world (58). Other postulated consequences of antenatal or infant vitamin D deficiency include growth faltering (59,60), type 1 diabetes (61), and asthma (62). However, rigorous studies of the broad health benefits of interventions to improve the antenatal or postnatal vitamin D status in South Asian mothers and infants have yet to be reported.

### Limitations

This study was limited by its small sample-size, restricted geographic scope, and cross-sectional design. We aimed to select control participants for the main case-control study in a manner that would enable inferences about the source population. Selection of control was necessarily non-random from the perspective of age and gender and, thus, unfortunately led to an over-representation of boys; however, there is unlikely to be a gender differential in vitamin D status during early infancy (63). Also, the requirement for an absence of reported history of ALRI probably produced negligible bias since only three otherwise eligible children were excluded for this reason. Aside from these caveats, the community-based sampling was likely random with respect to most determinants of vitamin D status. The group of infants aged 1-6 months was small but adequate to estimate the mean 25(OH)D within a 13-nmol/L range with 95% confidence. However, data were insufficient to yield precise estimates of the associations between infant and maternal characteristics and 25(OH)D. Another limitation was the lack of ancillary biochemical or radiographic data that may have revealed evidence of adverse consequences of low 25(OH)D. We did not report the findings of physical examinations because scoring systems for rickets are not very useful in early infancy, and a protocol for a standardized musculoskeletal clinical examination was not satisfactorily implemented. However, none of the toddlers had any clinical evidence of rickets according to the physician's examination (data not shown).

### Conclusions

This study provides initial observations on the vitamin D status of young infants in northeastern rural Bangladesh. However, it remains to be determined whether the relatively left-shifted distribution of 25(OH)D in this study sample is representative of the broader population and causally associated with an excess burden of rickets, symptomatic hypocalcaemia, growth faltering, or extra-skeletal health outcomes. Therefore, recommendations for universal antenatal and/or infant vitamin D supplementation in Bangladesh based on biochemical data alone would be premature. The causes and consequences of low 25(OH)D in young infants in South Asia must be further investigated.

## ACKNOWLEDGEMENTS

The authors thank the participants and caregivers for their kind cooperation and also thank the study field staff, the Projahnmo Sylhet team, and personnel at the Zakiganj subdistrict hospital. They particularly thank Kazi Moksedur Rahman (Deputy Executive Director, SHIMANTIK), Dr. Arun Kumar Roy (Project Research Physician, Projahnmo), Dr. Daniel Hossain (Project Research Manager, Projahnmo), Dr. Sirajul Islam (Upazila Health and Family Planning Officer), and Dr. Jonme Joy Dutta Shankar (Medical Officer, Zakiganj subdistrict hospital). The authors are grateful to Dr. Bruce Hollis and Dr. Carole Wagner (Medical University of South Carolina, USA), Dr. Samir K. Saha (Bangladesh Institute of Child Health, Dhaka Shishu Hospital, Dhaka, Bangladesh), and the Department of International Health at the Johns Hopkins Bloomberg School of Public Health. D. Roth was supported by training grants from the Canadian Institutes for Health Research and the Alberta Heritage Foundation for Medical Research.
